# *Aedes* spp. and Their Microbiota: A Review

**DOI:** 10.3389/fmicb.2019.02036

**Published:** 2019-09-04

**Authors:** Francesca Scolari, Maurizio Casiraghi, Mariangela Bonizzoni

**Affiliations:** ^1^Department of Biology and Biotechnology, University of Pavia, Pavia, Italy; ^2^Department of Biotechnology and Biosciences, University of Milano-Bicocca, Milan, Italy

**Keywords:** symbiosis, bacterial community, breeding sites, *Aedes* spp., vector control

## Abstract

*Aedes* spp. are a major public health concern due to their ability to be efficient vectors of dengue, Chikungunya, Zika, and other arboviruses. With limited vaccines available and no effective therapeutic treatments against arboviruses, the control of *Aedes* spp. populations is currently the only strategy to prevent disease transmission. Host-associated microbes (i.e., microbiota) recently emerged as a promising field to be explored for novel environmentally friendly vector control strategies. In particular, gut microbiota is revealing its impact on multiple aspects of *Aedes* spp. biology, including vector competence, thus being a promising target for manipulation. Here we describe the technological advances, which are currently expanding our understanding of microbiota composition, abundance, variability, and function in the two main arboviral vectors, the mosquitoes *Aedes aegypti* and *Aedes albopictus*. *Aedes* spp. microbiota is described in light of its tight connections with the environment, with which mosquitoes interact during their various developmental stages. Unraveling the dynamic interactions among the ecology of the habitat, the mosquito and the microbiota have the potential to uncover novel physiological interdependencies and provide a novel perspective for mosquito control.

## Introduction

Dengue is the most rapidly spreading vector-borne disease in the world with 2.5 billion people at risk and approximately 500,000 people developing severe dengue cases annually (World Health Organization [WHO], 2012). The increasing negative impact of dengue viruses on humans is partly associated with the range expansions of their primary vectors, *Aedes aegypti* and *Aedes albopictus*. Besides dengue viruses, *Ae. aegypti* and *Ae. albopictus* are also efficient vectors of Chikungunya, Zika, and other arboviruses, as well as dog heartworm and filarial nematodes (Bonizzoni et al., 2013).

Currently, control of mosquito populations is the only available strategy to prevent arboviral diseases because there are no therapeutic treatments for arboviruses and vaccines are limited.

Mosquitoes are holometabolous organisms with a life cycle involving two different types of habitats: larvae and pupae live in aquatic habitats, hereafter called “breeding sites,” and adults are subaerial (Clements, 2000). Only adult females transmit arboviruses, but controlling the juvenile stages is effective because significant reduction of larvae results in a decreased number of adults, thus reducing not only chances of disease transmission, but also nuisance. Consequently, a number of strategies have been developed to control larvae, including environmental sanitation, the use of insecticides or biological agents (McGraw and O’Neill, 2013). These conventional vector control methods are facing challenges because of their sustainability and organizational complexity. For instance, the Region Plan that was established in the Italian region of Emilia Romagna after the 2007 Chikungunya outbreak involved 280 municipalities and had a cost of 5.3 million euros over 3 years (Canali et al., 2017). Additionally, resistance to insecticides is emerging in natural *Ae. albopictus* populations and is widespread in *Ae. aegypti*, challenging the sustainability of this control measure (Xu et al., 2016; Moyes et al., 2017; Pichler et al., 2018). Thus, the development of novel, eco-friendly and easy to manage products or systems for vector control is urgently needed to complement traditional mosquito control methods.

Manipulation of mosquito microbiota is emerging as a promising field to develop novel vector control strategies. Examples that are already being implemented in the field include the use of entomopathogenic fungi such as *Beauveria bassiana*, which can be found on the water surface of breeding sites and kills larvae and adults of a number of mosquito species (Scholte et al., 2007; Farenhorst et al., 2009), and some strains of the alpha-proteobacteria *Wolbachia*, which induces cytoplasmic incompatibility and, when introduced into its not-natural host *Ae. aegypti*, it negatively impacts mosquito vector competence to dengue viruses (Saridaki and Bourtzis, 2010; Mohanty et al., 2016; O’Neill, 2018). Additional strategies aim at identifying natural symbionts of mosquitoes and either alter them genetically to express anti-pathogen effectors or disrupt their natural symbiosis with the insect host (Coutinho-Abreu et al., 2010; Ramirez et al., 2014; Kean et al., 2015; Saraiva et al., 2018a, b).

Here we provide an overview of the current knowledge on the composition, structure and function of *Aedes* spp. symbionts, with a focus on gut microbiota. We also highlight the technological progresses that are shaping our knowledge of mosquito microbiota and the exploitation of microbiota for vector control. The literature describing mosquito microbiota is ample and, in certain cases, controversial. The present review provides a summary of the available knowledge and may inadvertently omit some information. For these omissions, the authors apologize. For those interested in expanding on the topic, a number of helpful reviews have been published also in this Research Topic (see for example Minard et al., 2013a; Jupatanakul et al., 2014; Hegde et al., 2015; Wilke and Marrelli, 2015; Guégan et al., 2018b; Strand, 2018).

## Methodological Approaches to Study the Microbiota of *Aedes* spp.

**FIGURE 1:**
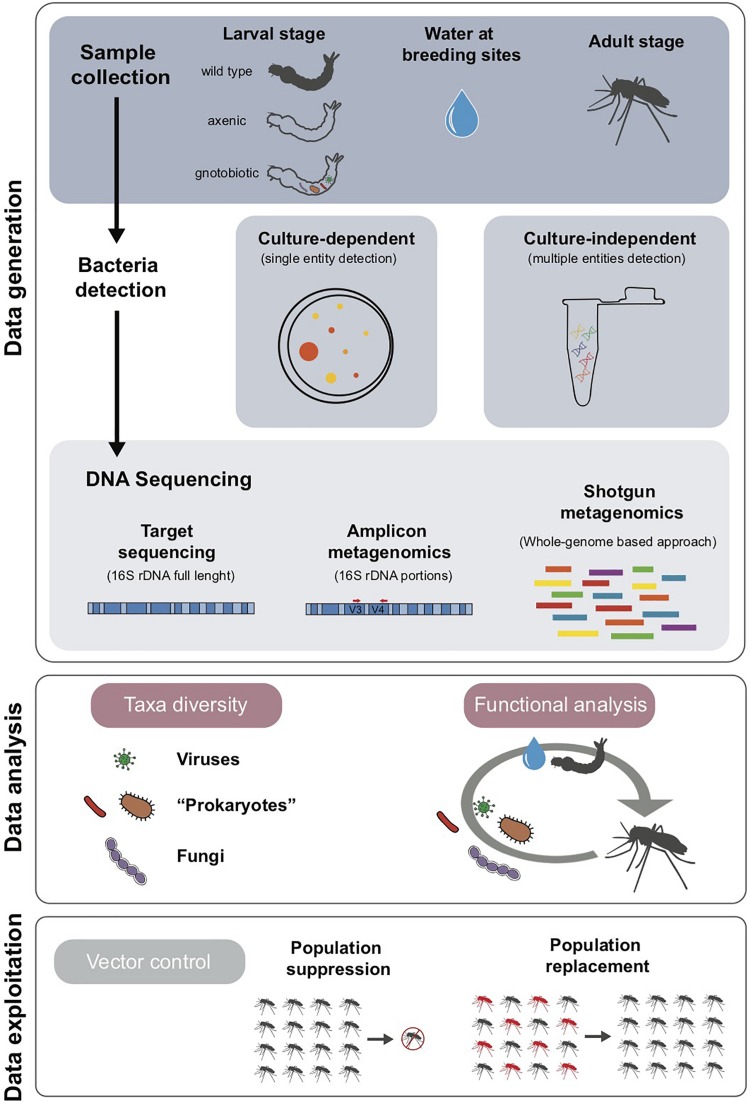
Workflow for microbiota analyses: data generation, analysis and exploitation.

The workflow for the study of *Aedes* spp. microbiota is organized in three main phases, i.e., data generation, analysis and exploitation, as summarized in Figure 1.

### Methods to Describe the Composition and Abundance of Microbiota in *Aedes* spp. Mosquitoes

The first paper that described the microbiota of *Ae. aegypti* was published in 2001 (Luxananil et al., 2001). In this study, the authors used a culture-dependent approach including isolation of *Ae. aegypti* guts of larvae collected from natural breeding sites in Thailand, plating of the homogenates onto Luria-Bertani (LB) agar plates and analysis of resulting colonies. Colonies were differentiated by morphology, as well as Gram-staining and standard biochemical assays (Koneman et al., 1992). In this first study, two strains of *Bacillus cereus* were identified as particularly abundant and, given the identified stable association with *Ae. aegypti*, authors suggested their potential exploitation for the development of mosquito larvicidal systems (Luxananil et al., 2001). A similar approach was adopted in a subsequent work focused on the characterization of the bacterial symbionts from *Ae. aegypti* crop (Gusmão et al., 2007). Besides identification of bacteria by morphology and biochemical approaches, authors also extracted the DNA and Sanger-sequenced their 16S rRNA gene to characterize them. *Serratia* sp. were the predominant bacteria in this tissue, and also *Bacillus* sp. and *Bacillus subtilis* were identified.

Culture-dependent methods were increasingly adopted in the following years based on different media and isolation techniques to investigate the composition and diversity of the microbiota in both *Ae. aegypti* and *Ae. albopictus* (Zouache et al., 2009; Chouaia et al., 2010; Gusmão et al., 2010; Apte-Deshpande et al., 2012; Ramirez et al., 2012; Valiente Moro et al., 2013; Minard et al., 2013b; Yadav et al., 2015, 2016; Charan et al., 2016). Culture-independent methods were also developed to overcome difficulties in recreating the physiological conditions necessary to cultivate bacteria. Culture-independent approaches include low-throughput techniques such as Denaturating Gradient Gel Electrophoresis (DGGE) (Chouaia et al., 2010; Zouache et al., 2011), taxonomic microarray hybridizations (Zouache et al., 2012), as well as more recent molecular strategies based on High Throughput DNA Sequencing technologies (HTS), such as DNA metabarcoding (or 16S rDNA amplicon sequencing) and metagenomics (Caporaso et al., 2010; Taberlet et al., 2012). These approaches allow researchers to achieve a more comprehensive and informative culture-independent picture of the bacterial communities that reside in mosquitoes (Osei-Poku et al., 2012; Minard et al., 2014; Pike et al., 2017; Guégan et al., 2018b).

The protocol for the amplification of 16S rDNA became a universal tool for determining the phylogenetic relationships among bacteria since its development in the early nineties (Weisburg et al., 1991; Patel, 2001). Nowadays, DNA metabarcoding is the most common sequencing approach to characterize the microbial community in a sample (Pollock et al., 2018). This method is based on the amplification and sequencing of hypervariable region(s) of the 16S rDNA, nowadays most-frequently using Illumina technology, primarily the MiSeq system, to achieve the most accurate longest reads with high throughput. Variability of the 16S rDNA is usually high enough to allow accurate *taxa* characterization but may not always allow unambiguous identification at a lower classification level such as genus or species. DNA metabarcoding has been applied to both *Ae. aegypti* and *Ae. albopictus* (Table 1), allowing to analyze samples in a very cost-efficient manner.

**TABLE 1:** Studies analyzing *Aedes aegypti* and *Ae. albopictus* microbiota using 16S rRNA gene metabarcoding approaches.

**Mosquito species**	**Mosquito origin^1^**	**Dev. stage**	**Tissue**	**Methodological approach^2^**	**References**
***Aedes aegypti***					
	Kilifi (Kenya)	AF	M	V3 of 16S rRNA gene/Roche 454 FLX	Osei-Poku et al., 2012
	UGAL strain	L; BS; AF; ConR/STR	WB	V1-V2 of 16S rRNA gene/Roche 454 GS-J	Coon et al., 2014
	Vila Valqueire (Brazil) strain	AF	M	V3-V5 of 16S rRNA gene/Roche 454 GS-J	David et al., 2016
	Jacksonville (FL, United States) UGAL strain	L	WB	V3-V4 of 16S rRNA gene/Illumina MiSeq	Coon et al., 2016b
	Babinda (Australia) *Wolbachia*-infected *w*Mel line	AF	WB	V3-V4 of 16S rRNA gene/illumina MiSeq	Audsley et al., 2017
	Gabon (Africa)	BS; AF	M	V5-V6 of 16S rRNA gene/Illumina MiSeq	Dickson et al., 2017
	Houston (TX, United States) Galveston strain	SF-AF	WB	V3-V4 of 16S rRNA gene/Illumina MiSeq	Hegde et al., 2018
	Cairns (Australia); Phnom Penh (Cambodia); Cayenne (French Guiana); Bakoumba (Gabon); Saint Francois (Guadaloupe); Zika (Uganda)	AF	M	V5-V6 of 16S rRNA gene/Illumina MiSeq	Dickson et al., 2018
	Cairns (Australia)	AF; L	WB	16S rRNA gene/Illumina MiSeq	Audsley et al., 2018
	Rockefeller strain	AF	M	V3-V4 of 16S rRNA gene/Illumina MiSeq	Muturi et al., 2019
	PP-Campos (Brazilian strain)	AF	WB	V3-V4 of 16S rRNA gene/Illumina MiSeq	Villegas et al., 2018
	New Orleans, LA 2011 strain	SF-AF; AM	Fo + M; SG; RO	V4 of 16S rRNA gene/Illumina MiSeq	Mancini et al., 2018
	Nakhon Nayok (Thailand)	AF	WB	V3 of 16S rRNA gene and 18S rRNA Roche 454 FLX	Thongsripong et al., 2017
***Aedes albopictus***					
	Toamasina (Madagascar)	NBF-AF	WB	V5-V6 of 16S rRNA Roche 454 FLX Titanium	Minard et al., 2014
	Ho Chi Minh City, Binh Du’o’ng, Vung Tau City, Bu Gia Map (Vietnam); Saint-Priest, Portes-Lès-Valence, Nice (France)	AF	M	V5-V6 of 16S rRNA gene/Illumina MiSeq	Minard et al., 2015
	Athens (GA, United States) CDC strain	L	WB	V3-V4 of 16S rRNA gene/Illumina MiSeq	Coon et al., 2016b
	Champaign County (IL, United States)	AF	M	V3-V5 of 16S rRNA gene/Illumina MiSeq	Muturi et al., 2017
	Guangzhou (China) Foshan strain	BS, L (3rd instar), P, A	WB	V4 of 16S rRNA gene/Illumina MiSeq	Wang et al., 2018
	Houston (TX, United States) Galveston strain	SF-AF	WB	V3-V4 of 16S rRNA gene/Illumina MiSeq	Hegde et al., 2018
	Trento (Italy)	AF	M	V5-V6 of 16S rRNA gene/Illumina MiSeq	Rosso et al., 2018
	MRA-804 strain	SF-AF; AM	Fo + M; SG; RO	V4 of 16S rRNA gene/Illumina MiSeq	Mancini et al., 2018
	Nakhon Nayok (Thailand)	AF	WB	V3 of 16S rRNA gene and 18S rRNA Roche 454 FLX	Thongsripong et al., 2017

*AF, adult females; L, 4th instar larvae; BS, breeding site water; ConR, eggs laid by conventionally-reared females; STR, eggs laid by blood fed females emerging from surface-sterilized pupae; SF, sugar fed; AM, adult males; NBF, non-blood fed; P, pupae; A, adults; M, midgut; WB, whole body; Fo, foreguts; SG, salivary glands; RO, reproductive organs. ^1^When field mosquitoes were used, sampling site is cited. ^2^rDNA region amplified and Sequencing Platform.*

More recently, Shotgun Metagenomic Sequencing (SMS) was implemented through HTS. This approach does not rely on an initial PCR step with universal primers (for instance targeting bacterial 16S rDNAs), thus allowing to extend the analyses of insect microbiota beyond bacteria to fungi and viruses and allowing bacteria identification beyond the 16S rRNA genes (Warnecke et al., 2007; Runckel et al., 2011; Engel et al., 2012). SMS was initially applied to identify viruses infecting wild mosquitoes, including those of the *Culex*, *Anopheles, Ochlerotatus*, and *Aedes* genera (Ma et al., 2011; Ng et al., 2011; Cook et al., 2013; Chandler et al., 2014, 2015; Xia et al., 2018). SMS was used to analyze *Ae. aegypti* and *Ae. albopictus* strains artificially infected with dengue virus type 1 and 3 (DENV-1 and DENV-3), chikungunya (CHIKV), or yellow fever (YFV) viruses (Bishop-Lilly et al., 2010; Hall-Mendelin et al., 2013). The results of these studies showed that SMS has the potential to be integrated in the framework of arbovirus surveillance programs, with the advantages of obviating the need for culture-based approaches and prior knowledge of etiologic agents (Bishop-Lilly et al., 2010). This is possible since DNA and RNA viruses can be detected in mosquito blood meal for up to 24 h after initial ingestion (Grubaugh et al., 2015). Despite the power of SMS, this technique has not been extensively applied to *Aedes* spp.

Culturomics recently emerged as a novel tool to discover still unknown microbes (Lagier et al., 2018). This method consists in the combination of multiple culture conditions using a high-throughput approach (i.e., use of different selective and/or enrichment culture conditions) followed by matrix-assisted laser desorption/ionization-time of flight (MALDI-TOF) or 16S rDNA amplification and sequencing to identify the growing colonies. This method was applied to *Anopheles gambiae*, *Culex quinquefasciatus*, and *Ae. albopictus* to characterize the bacterial diversity of mosquito midguts (Tandina et al., 2016). With this approach, 17 previously unknown bacterial species were identified in *An. gambiae*, suggesting the potential of culturomics for expanding our knowledge of the microbiota composition. The advantages provided by culturomics include the ability to detect minor community members, the capacity to provide information about the viability of the detected symbionts, and the potential for further improvements due to innovations in automation and miniaturization (Greub, 2012).

Protocols to produce axenic individuals (i.e., bacteria-free) or gnotobiotic larvae (i.e., larvae colonized by a single bacterial species or a simplified bacterial community) were generated to study the physiological impact of the microbiota. Early studies on the functions of microbiota in *Ae. aegypti* were based on the use of sterile conditions and diet supplementation with vitamins and nutrients (Lang et al., 1972). More recent studies used a combination of ethanol and bleach to sterilize the egg surface and standard larval food, previously sterilized by irradiation (Coon et al., 2014, 2016a). Gnotobiotic larvae are generated by inoculation of a given bacterial isolate in flasks containing sterile water, sterilized standard diet and the axenic first instar larvae (Coon et al., 2014, 2016a). Interestingly, despite the use of antibiotic treatments to manipulate insect bacterial communities, a recent study clearly indicated that several antibiotics failed in achieving the full elimination of bacteria in *Ae. aegypti* (UGAL strain) and showed adverse effects on the fitness of first instars larvae (Coon et al., 2016b). An alternative approach to rear axenic adult mosquitoes was recently described based on maintaining larvae hatched from surface-sterilized eggs on agar plugs containing yeast and liver extract (Correa et al., 2018). This method was also used for the production of adult mosquitoes with simplified microbiota (i.e., from one to three symbiont species) (Correa et al., 2018).

### Methods to Describe the Composition and Abundance of Microbiota of *Aedes* spp. Breeding Sites

Concerns about the microbial quality of drinking water together with the increasingly recognized importance of free-living and host-associated microbes to the function of both the ecosystems and living organisms greatly stimulated the development of protocols for the analysis of the microbiota in aquatic environments (Jackrel et al., 2017). The complexity of aquatic environments requires the adoption of integrated analytic systems in which stringent water filtration methods, HTS technologies and bioinformatics are combined to cope with the low concentrations of organisms in aquatic environments (Bruno et al., 2017) and with the ultrasmall cell size of some aquatic bacteria (Brown et al., 2015; Luef et al., 2015). Such integrated approaches began to be applied to isolate and characterize the bacteria present in *Aedes* spp. larval breeding sites, showing that a substantial fraction of the microbiota in mosquitoes is acquired through larval feeding in breeding sites (Coon et al., 2014, 2016b; Dada et al., 2014; Dickson et al., 2017; Wang et al., 2018). Most analyses involve water filtration allowing to retain microorganisms >0.2 μm in size (Bruno et al., 2018). Recently, novel and more stringent protocols were developed, which, through serial water filtration with membrane filters of decreasing pore sizes, allow to collect and concentrate the bacterial samples in the water. Such methods make use of tangential flow filtration (TFF) systems combined with filtration modules able to retain particles <0.1 μm in size, thus allowing to physically separate macro-organisms from micro-organisms and viruses. DNA extracted from these water samples is then sequenced using HTS approaches (Bruno et al., 2016, 2017, 2018). Studies in *Anopheles* spp. mosquitoes (Gimonneau et al., 2014) showed that the depth in which breeding site water is sampled may influence the composition of bacteria. *Aedes* spp. mosquitoes tend to breed in small, often human-made and not stable breeding sites, for which there should be no depth differences (Dickson et al., 2017).

## The Microbiota of *Aedes* Mosquitoes: Origin and Composition

### Microbiota and Mosquito Habitat

Depending on their life-stage, mosquitoes interact with microbiota differently. At the larval stage, *Aedes* spp. microbiota is acquired primarily through feeding in breeding site water. In both *Ae. aegypti* and *Ae. albopictus*, the composition of larval microbiota represents a subset of the Operational Taxonomic Units (OTUs) found in the breeding site water (Coon et al., 2016b). The lower abundance of bacterial *taxa* in the larvae as compared to what found in breeding site water suggests that bacteria that establish symbiosis early during larval development may inhibit the colonization by additional *taxa* (Ponnusamy et al., 2008; Dada et al., 2014). The composition of larval microbiota varies greatly among sites, but strong similarities are found among larvae of different species that breed in the same site (Coon et al., 2016b). These data support the relevance of larval habitat in shaping *Aedes* spp. microbiota. Distinct bacterial communities were identified between domestic and sylvatic *Ae. aegypti* habitats further supporting the important effect of the ecological context of the breeding sites in defining the composition of mosquito microbiota (Dickson et al., 2017).

Adults can acquire bacteria from their breeding water while they emerge from their pupal cases, as shown for *An. gambiae* (Lindh et al., 2008). In addition, mosquitoes have been proposed to be able to transmit symbionts back to the breeding sites while laying eggs, thus affecting the microbial community which larvae are exposed to, and supporting a certain level of vertical transmission (Coon et al., 2016b). This is particularly important for *Ae. aegypti* and *Ae. albopictus*, which are able to exploit small and temporary water containers as larval breeding sites. In these conditions, water biogeochemical properties such as pH and concentration of ions, temperature, food sources and microorganism abundance may undergo sharp and rapid variations, which affect microbial composition. For instance, in microcosm-based experiments performed with *Ae. triseriatus*, the presence of larvae in the water was shown to contribute to create enriched and anoxic conditions which favored the growth of Enterobacteriaceae (Kaufman et al., 1999). Besides influencing the composition of larval gut microbiota, the microbiota of the breeding site also plays a role in mediating attraction and oviposition responses of mated *Aedes* spp. females. For example, *Bacillus cereus* and *Pseudomonas aeruginosa* elicit oviposition responses in *Ae. aegypti* (Hasselschwert and Rockett, 1988). Similarly, *Ae. aegypti* females were shown to be significantly induced to oviposit in water containing a suspended solution of *Acinetobacter calcoaceticus* (Benzon and Apperson, 1988). In *Ae. albopictus*, *Psychrobacter immobilis* isolated from the water of larval breeding sites elicited higher oviposition responses from gravid females than did water deprived of that bacterial species (Trexler et al., 2003).

The variable nature of aquatic environments, including fluctuations in temperature, pH and oxygen content that impact microbial growth, prevents mosquitoes from reliably encountering particular and standard bacterial species and support the hypothesis of a dynamic host-symbiont interaction (Zouache et al., 2011; Osei-Poku et al., 2012; Minard et al., 2013a; Valiente Moro et al., 2013; Coon et al., 2016b).

Despite habitat-related differences in the composition of larval microbiota were observed, a number of bacterial *taxa* have been consistently found in all tested *Aedes* spp. and have been proposed to constitute the ‘core microbiota’ of mosquitoes (Walker et al., 1991; Ponnusamy et al., 2008; Yee et al., 2012; Dada et al., 2014; Dickson et al., 2017; Guégan et al., 2018b). *Aedes* spp. microbiota is composed primarily by Gram-negative aerobic and facultative-anaerobic bacteria, as generally occurring in other mosquito species (Wang et al., 2011; Boissière et al., 2012; Osei-Poku et al., 2012; Coon et al., 2014, 2016a,b; Gimonneau et al., 2014; Duguma et al., 2015; Muturi et al., 2016b; Valzania et al., 2018). Only two obligate anaerobe *taxa* have been detected in *Aedes* spp. so far. These anaerobe *taxa* are *Clostridium*, found in *Ae. aegypti* (Coon et al., 2014), and *Blautia*, detected in *Ae. albopictus* (Minard et al., 2014). Members of the *Blautia* genus were previously isolated from mammalian gut and were suggested to play a role in nutrient assimilation (Bernalier et al., 1996; Gagen et al., 2010; Eren et al., 2015).

### Microbiota of Different Mosquito Developmental Stages

Microbiota composition changes during the development from larvae to adults (Thiery et al., 1991; Vazquez-Martinez et al., 2002; Rani et al., 2009; Dinparast Djadid et al., 2011; Wang et al., 2011; Chavshin et al., 2012). Adults eliminate larval midgut bacteria during metamorphosis, for instance *Ae. aegypti* expel more than 90% of the bacterial species during molting and metamorphosis (Moll et al., 2001; Moncayo et al., 2005; Wang et al., 2018), similarly to what is observed in other insects (Wang et al., 2011; Martinson et al., 2012; Junqueira et al., 2017). Nevertheless, several bacteria are *trans*-stadially transmitted and bacteria may be acquired also during blood feeding, as reported for the arboviral vector *Culicoides imicola* (Moll et al., 2001; Coon et al., 2014; Díaz-Sánchez et al., 2018).

Actinobacteria and Bacteroidetes, members of Proteobacteria, were found to be consistently present in 4th instar larvae of both *Ae. aegypti* and *Ae. albopictus* (Coon et al., 2014, 2016a,b; Audsley et al., 2017; Wang et al., 2018). Other genera that are frequently found in the gut of larvae include *Chryseobacterium*, *Elizabethkingia*, *Pseudomonas*, *Nisseria*, and *Enterobacter* (DeMaio et al., 1996; Dong et al., 2009; Chouaia et al., 2010; Cirimotich et al., 2011; Dinparast Djadid et al., 2011; Oliveira et al., 2011; Wang et al., 2011; Osei-Poku et al., 2012; Bahia et al., 2014). The Actinobacteria *Leucobacter* and *Microbacterium*, both belonging to *Microbacteriaceae* family, are abundant in *Ae. aegypti* larvae, but nearly absent in adults (Coon et al., 2014). In contrast, *Chryseobacterium* (Flavobacteriaceae) was a common component of mosquito microbiota at all life-stages (Coon et al., 2014).

In the case of adult mosquitoes, Proteobacteria, Bacteroides, Firmicutes, and Actinobacteria are the phyla grouping more than 99% of the total microbiota community components (Mancini et al., 2018). More specifically, members of *Enterobacteriaceae* (e.g., *Enterobacter*, *Klebsiella*, *Kluyvera*), *Erwiniaceae* (e.g., *Pantoea*), *Yersiniaceae* (e.g., *Serratia*), *Acetobacteraceae* (e.g., *Asaia)*, *Enterococcaceae* (e.g., *Enterococcus*), and of *Bacillaceae* (e.g., *Bacillus*) are the most-frequently described bacteria from the gut of adult *Aedes* spp. (DeMaio et al., 1996; Pumpuni et al., 1996; Straif et al., 1998; Fouda et al., 2001; Gonzalez-Ceron et al., 2003; Lindh et al., 2005; Favia et al., 2007; Terenius et al., 2008; Crotti et al., 2009; Dong et al., 2009; Rani et al., 2009; Gusmão et al., 2010). Characterization of microbiota is biased by the technique used and the level of variability within the 16S rRNA genes. Thus, while higher *taxa* assignment is certain, lower classification may be problematic and lead to contrasting results.

### Microbiota of Different Body Tissues

**FIGURE 2:**
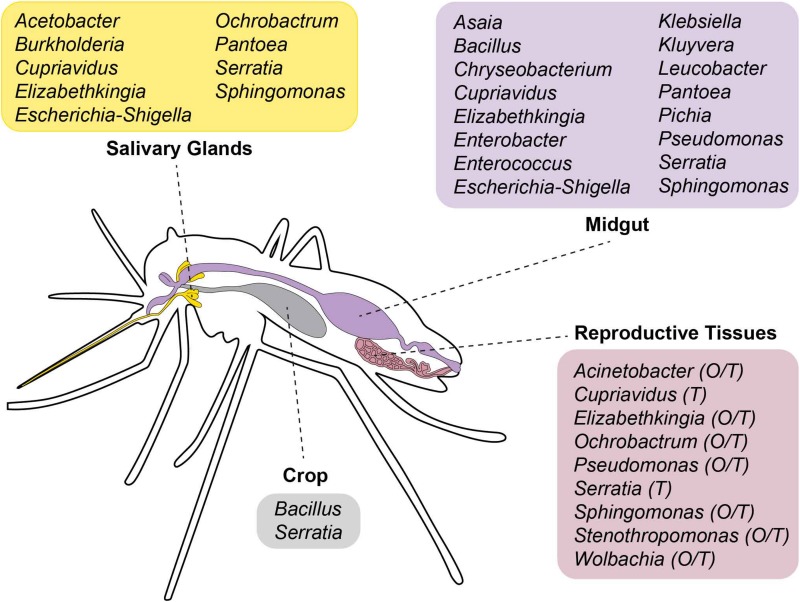
Scheme showing the tissue localization of bacterial genera so far identified in *Aedes* spp. mosquitoes. O, ovaries; T, testes.

Most studies focus on the microbiota of the gut because of its direct implications with mosquito vector biology (Dharne et al., 2006). However, microorganisms can colonize other organs, including reproductive tissues and salivary glands, both in *Anopheles* (Sharma et al., 2014; Tchioffo et al., 2016) and *Aedes* spp. mosquitoes (Mancini et al., 2018). An overview of the bacterial genera so far identified in *Aedes* spp. tissues is reported in Figure 2.

In both *Ae. aegypti* and *Ae. albopictus* adults of different laboratory strains, the phylum Proteobacteria is dominant in gut, ovaries, salivary glands, testes and male accessory glands, with tissue-specific tropism being detected (Mancini et al., 2018). For instance, the microbiota of reproductive organs of both sexes appears more diverse than that colonizing either the gut or salivary glands. Inter-specific differences in the tissue distribution of the microbiota were also detected. For instance, while Alphaproteobacteria represent the 97% of female and the 73% of male total microbiota in reproductive tissues of *Ae. albopictus*, they constitute about 30% of the total bacteria in reproductive tissues of *Ae. aegypti*. *Wolbachia* dominates in *Ae. albopictus* ovaries (94%), while it co-exists with bacteria of the genera *Sphingomonas*, *Cupriavidus*, and *Serratia* in testes. Gammaproteobacteria are the dominant taxon in salivary glands of female *Ae. albopictus*, while the microbiota of *Ae. aegypti* salivary glands is richer and includes representatives of the genera *Serratia, Escherichia–Shigella*, *Pantoea*, *Acetobacter*, *Sphingomonas*, *Burkholderia*, and *Cupriavidus*.

In the gut of both *Aedes* species, Alpha-, Beta- and Gammaproteobacteria are equally represented, with *Sphingomonas*, *Asaia*, *Cupriavidus*, *Escherichia-Shigella*, *Pseudomonas* and *Serratia* being the most-frequent *taxa*. Sex differences in the composition of the microbiota are detected, with the dominance of Alphaproteobacteria in male guts (Mancini et al., 2018). Whether the composition of *Aedes* spp. microbiota is richer than what is found in other species is still controversial (Dickson et al., 2018; Mancini et al., 2018). A number of factors, such as the number of generations in the insectary, the age and the genetic background of the species studied, along with the rearing conditions may influence microbiota composition.

### Microbiota of Wild and Laboratory Mosquitoes

Microbiota composition was analyzed in both laboratory strains and wild populations. Laboratory strains include UGAL, Galveston, Rockefeller and MOYO for *Ae. aegypti* and CDC, Foshan and Galveston for *Ae. albopictus* (Charan et al., 2013) (see Table 1). Field mosquitoes were sampled across the global distribution of both species (Kamal et al., 2018). Most of the studies were performed on adult female midguts, followed by 4th instar larvae and their breeding site water.

Upon laboratory colonization, the composition of adult midgut microbiota among different strains derived from distinct *Ae. aegypti* geographic populations was highly similar at the phylum level (Dickson et al., 2018). The landscape of the overall microbiota across strains does not change even in presence of differences in the abundance of specific *taxa* within each phylum (Charan et al., 2013; Short et al., 2017). These results pose the challenging question of whether preferential associations between *Aedes* spp. genotypes and specific bacterial *taxa* exist or are selected for during laboratory colonization.

*Aedes* spp. sampled in different areas showed a limited diversity within bacterial communities at high taxonomic levels (i.e., phylum and family) (Valiente Moro et al., 2013; Minard et al., 2014; Thongsripong et al., 2017), similarly to data reported for field-collected *Anopheles* spp. (Boissière et al., 2012; Osei-Poku et al., 2012). *Micrococcus* of the Actinobacteria phylum, *Staphylococcus* of the Firmicutes phylum, and primarily members of the Proteobacteria phylum, such as *Comamonas*, *Acinetobacter*, *Enterobacter*, and *Pseudomonas* genera, are consistently present in wild mosquitoes (Ramirez et al., 2012; Audsley et al., 2017, 2018). Proteobacteria include Gram-negative bacteria that have been suggested to be abundant in insects due to a more effective capacity to invade and proliferate within new hosts and/or their active recruiting by insects (Jones et al., 2013). Similarly, *Ae. albopictus* samples from Northern Italy (Trentino region) display a lower degree of microbial diversity when compared to French and Vietnamese populations (Rosso et al., 2018). Whether this result is dependent on differences in breeding sites water or the fact that populations from Italy and France are new populations is an open question. The absence of a relation between population genetic origin and midgut microbiota was recently shown in two *Ae. albopictus* populations from tropical (La Réunion) and temperate (Montpellier, Continental France) regions reared under controlled laboratory conditions (Minard et al., 2018). These contrasting data highlight the need to continue investigating the impact of *Aedes* spp. population dynamics on microbiota composition.

One common trait revealed by several studies is the high inter-individual variability in the composition of the microbiota, especially at a lower classification level (Boissière et al., 2012). Specific OTUs may be found exclusively in one specimen, and individual OTUs may represent over 90% of the microbiota of one mosquito (Wang et al., 2011; Osei-Poku et al., 2012; Rosso et al., 2018). This aspect is particularly relevant since it may be related to variations in vector competence as well as mate choice, as occurring in the fruit fly *Drosophila melanogaster*, where flies prefer mates sharing a similar bacterial gut community (Sharon et al., 2010).

## Factors That Shape *Aedes* spp. Microbiota

Habitat-related parameters affect bacterial communities, thus impacting the composition of mosquito microbiota. For example, increase of water temperature in breeding sites results in higher abundance of Betaproteobacteria and this correlates with higher abundance of *Anopheles* vs. *Aedes* spp. larvae (Hörtnagl et al., 2010; Onchuru et al., 2016). Anthropogenic activities also impact the bio-geochemical properties of breeding sites, and, in turn, affect mosquito microbiota. Fertilizers containing ammonium and phosphorous contaminating aquatic habitats are known to affect the development and abundance of bacteria (Muturi et al., 2016b), which are a nutrient source for Culicidae larvae (Merritt et al., 1992). The increasing abundance of residual antibiotics in the environment has been shown to impact the composition of the microbiota to which mosquitoes are exposed. Antibiotics in human blood disrupt gut microbiota of *An. gambiae* females and enhance susceptibility to *Plasmodium* spp. infection (Gendrin et al., 2015). In addition, larval ingestion of antibiotics alters bacterial composition in *Ae. albopictus* adults, with reduction or even elimination of specific *taxa* and concomitant proliferation of *Wolbachia* and *Dysgonomonas* spp. (Guégan et al., 2018a). *Dysgonomonas* spp. is abundant in *Ae. albopictus* populations and is known to produce vitamin B12 in termites (Husseneder et al., 2009; Minard et al., 2015). Whether this bacterium plays a similar role in *Ae. albopictus* remains to be determined.

Host-related factors, including nutrition, development and sex, also influence microbiota composition (Minard et al., 2013a). For instance, blood-meal induces an overall decrease of OTU numbers with an increase in relative abundance of bacteria of the genera *Chryseobacterium* and *Delftia* in *Ae. aegypti*, and blood- and sugar-fed females harbor distinct bacterial communities (Oliveira et al., 2011; Wang et al., 2011; Coon et al., 2014; Yadav et al., 2016). Changes in the composition of the gut microbiota following a blood meal may be due to the oxidative stress associated with the catabolism of the blood meal, as proposed for *An. gambiae* (Wang et al., 2011; Terenius et al., 2012). The two above-mentioned dominant genera were also detected on the surface of eggs, supporting the idea of vertical transmission (Coon et al., 2014). The source of blood meal may also influence the composition of the microbiota in mosquito midguts, similar to what occurs in *Ixodes pacificus* ticks (Swei and Kwan, 2017; Muturi et al., 2019). In particular, members of the genera *Leucobacter*, *Chryseobacterium*, *Elizabethkingia*, and *Serratia* were characteristic of either newly emerged *Ae. aegypti* or adults fed on chicken, rabbit, and human blood, respectively (Muturi et al., 2019). Sugar-fed mosquitoes displayed higher abundance of *Pseudomonas* spp. and unclassified Acetobacteraceae, which were previously found in associations with insects relying on sugar-based diets (Crotti et al., 2010; Muturi et al., 2016a). While blood directly goes to the midgut, sugar meals are stored in the crop as food reserves (Clemens, 1992). The crop of *Ae. aegypti* harbor bacteria including *Serratia* (Yersiniaceae) and the yeast *Pichia* sp., which can be transferred to the midgut along with food (Gusmão et al., 2007, 2010). *Elizabethkingia* spp. (Flavobacteriaceae) was found only in sugar-fed females (David et al., 2016), in agreement with findings in *An. gambiae*, where *Elizabethkingia* spp. abundance was reduced after blood feeding (Wang et al., 2011). The different nutritional behavior of male and female mosquitoes may contribute in the observed sex-related differences in the composition of microbiota (Zouache et al., 2011; Minard et al., 2018). Such differences may also relate to the different tropism of endosymbionts for female and male reproductive organs, as observed in *Anopheles* spp. (Segata et al., 2016).

The composition of microbiota also changes with mosquito age, probably as the result of interspecific competition among symbionts for sugar sources (Dong et al., 2009; Terenius et al., 2012), or, as occurring in other insects, nutrient availability in the gut or mosquito immunity (Hillyer et al., 2005; Montagna et al., 2015).

The importance of bacteria interactions, as well as potential interaction with viruses, is becoming more and more evident as a regulator of the composition and abundance of the microbiota and has practical implications that we describe below. Microbiota composition of *Ae. aegypti* adults changes following ZIKV infection, with Rhodobacteraceae and Desulfuromonadaceae emerging as biomarkers of ZIKV infection (Villegas et al., 2018). When stable symbiosis is artificially established in *Ae. aegypti*, *Wolbachia* dynamically interacts with other members of the microbiota community but has minimal effects on microbiota composition (Audsley et al., 2017).

## Physiological Impacts of the Microbiota

Studies aiming at clarifying patterns of co-occurrence and co-exclusion among the components of the microbiota are being perused to decipher the physiological impact of the microbiota and shed light on complex phenotypes. For example, *Serratia* and *Cedecea* spp. displayed several co-exclusionary relationships with dominant *taxa* such as members of the genera *Asaia*, *Pseudomonas*, and *Enterobacter* in the microbiota of *Ae. aegypti*, *Ae. albopictus*, and *C. quinquefasciatus* from both the field and the laboratory (Hegde et al., 2018). Additionally, the first whole genome metagenomic analysis of *An. albimanus* revealed links between microbiota and phenotypic resistance to the insecticide fenitrothion, suggesting a role of microbiota in insecticide resistance (Dada et al., 2018).

*Aedes aegypti* mosquitoes were initially thought to require living bacteria for development, as axenic larvae die as first instars differently than for *Anopheles* spp. (Chouaia et al., 2012). This interaction did not appear to depend on a particular bacterial species or community assemblage, as several different bacterial species rescued development of gnotobiotic larvae (Coon et al., 2014). In such gnotobiotic mosquitoes, each of the individual bacteria tested proliferated in absence of other community members, with the exception of *Microbacterium* and *Leucobacter* spp. These last *taxa* require other bacteria to survive in *Ae. aegypti*. Taken together, these results suggest that several members of the larval gut microbiota support development and egg production comparably to conventionally reared individuals with a mixed bacterial community (Coon et al., 2016a).

As described above, survival of axenic larvae may be achieved under specific conditions. In contrast to previous experiments, Correa et al. (2018) managed to rear axenic larvae to adulthood by providing high concentrations of liver and yeast extract in a semi-solid form. Axenic larval developmental time was longer than that of larvae with an unaltered microbiota. These data support the idea that the primary symbiotic association between gut bacteria and *Ae. aegypti* is essentially nutritional, as live bacteria and fungi do not appear to be essential to mosquito development.

Adult gut microbiota affects blood meal digestion. Treatment with antibiotics reduced the abundance of culturable gut bacteria, resulting in slower digestion of the blood bolus and statistically significant reductions in the number of laid eggs (Gaio et al., 2011). *Enterobacter* and *Serratia*, in particular, are involved in hemolytic activity (Coon et al., 2016a).

Exposure to bacteria during larval development affects adult traits related to pathogen transmission, suggesting that a better understanding of larval ecology has the potential to reveal determinants of pathogen transmission by *Aedes* spp. (Dickson et al., 2017). Earlier studies in *Ae. aegypti* showed that removal of the gut microbiota with antibiotics increases mosquito susceptibility to DENV-2 infection (Xi et al., 2008), and that *Serratia odorifera* is able to enhance DENV-2 susceptibility (Apte-Deshpande et al., 2012). The increase in DENV loads and prevalence correlate with the presence of *Serratia* because *Serratia* secretes SmEnhancin, a protein that cleaves off membrane-bound mucins and weakens the peritrophic matrix favoring viral dissemination out of the midgut (Wu et al., 2019). This effect on viral dissemination was not observed when other 20 commensal bacteria were tested, supporting the idea of a species-specific effect of the microbiota on *Ae. aegypti* vector competence (Wu et al., 2019). *Serratia*-positive mosquitoes were obtained from DENV endemic regions, while *Serratia*-negative mosquitoes were caught in non-DENV-endemic regions supporting the hypothesis that microbiota composition may contribute to the observed differences in vector competence across *Ae. aegypti* populations (Souza-Neto et al., 2019).

## Beyond Bacteria

The bacterial component of mosquito microbiota is by far the most widely investigated. However, *Aedes* spp. microbiota comprise also other entities such as non-pathogenic fungi, pathogenic yeasts and viruses (Guégan et al., 2018b).

Less than five fungal species were identified in *Ae. aegypti* and *Ae. albopictus* (Bozic et al., 2017). Whether this limited number is indicative of a streamlined fungal community remains to be determined. *Penicillium* was found in wild adults and larvae based on the morphological analysis of fungal colonies (da Costa and de Oliveira, 1998). Subsequently, a combination of culture-dependent methods and PCR amplification of the 28S rRNA gene (i.e., 28rRNA and 16rRNA amplification are analogous as fungi do not have 16 rRNA) allowed the identification of *Pichia* in the crop of newly emerged unfed females of the Rockefeller strain (Gusmão et al., 2007). Members from the genus *Pichia* and *Candida* were also found in the midgut and in midgut and ovaries of *Ae. aegypti* mosquitoes, respectively (Frants and Mertvetsova, 1986; Gusmão et al., 2010). *Pichia* was isolated only from sugar-fed females (Gusmão et al., 2010). By using culture-dependent method, *Candida parapsilosis* and *Meyerozyma guilliermondii* were identified in larvae, pupae and adults (in both gut and gonads) of *Ae. aegypti* and *Ae. albopictus* laboratory strains and wild-collected adults from Brazil, Bangladesh and Italy (Bozic et al., 2017). *Candida parapsilosis* and *Meyerozyma guilliermondii* can become opportunistic human pathogens under specific physiological conditions (Singh and Parija, 2012; Tan et al., 2016). The identification of these fungi in *Aedes* spp., which complete their life cycle in anthropized environments, suggests these mosquitoes could contribute to the dissemination of pathogenic yeasts, thus increasing their public health relevance (Bozic et al., 2017). *Meyerozyma guilliermondii* colonizes the guts of insects from several taxa (Stefanini, 2018); for instance, it is the dominant species in the mycobiota of the leishmaniasis vector *Phlebotomus perniciosus* where it was proposed to contribute in uric acid degradation (Martin et al., 2018). Metabolic interactions between members of the mycobiota and the mosquito host are being discovered. As an example, a fungus from the *Talaromyces* genus was identified to be naturally present in the midgut of field-caught *Ae. aegypti* females from Puerto Rico (Angleró-Rodríguez et al., 2017) using a combination of microscopy and sequencing of the rRNA internal transcribed spacer (ITS) (Schoch et al., 2012). *Talaromyces* was found to enhance DENV2 infection by transcriptional and enzymatic inhibition of trypsins in the midgut, thus increasing mosquito vector competence (Angleró-Rodríguez et al., 2017).

Mosquito virome includes arthropod-borne viruses (i.e., arboviruses) able to replicate in mosquitoes and vertebrates, and recently identified insect-specific viruses (ISVs), which are restricted to insects and do not replicate in vertebrates (Braack et al., 2018; Öhlund et al., 2019).

Metagenomic approaches were initially used for discovery and surveillance of specific viruses, such as DENV-1 and Phasi Charoen-like virus (PCLV) in *Ae. aegypti* (Bishop-Lilly et al., 2010; Chandler et al., 2014) and CHIKV, DENV-3 and YFV in *Ae. albopictus* (Hall-Mendelin et al., 2013). The first study using mosquito virus metagenomic sequencing to describe the diversity of DNA viruses was performed on wild mosquitoes from California (Ng et al., 2011). This study analyzed pools of female mosquitoes from different species collected in three geographical sites, comprising *Culex erythrothorax* as well as other undetermined species. This study revealed that the viral community was highly diverse across samples and most of its members were uncharacterized. The identified viral sequences showed similarity to members of the Anelloviridae, Circoviridae, Herpesviridae, Poxviridae, and Papillomaviridae families, which infect mammals and birds (Ng et al., 2011). This study also showed for the first time that mosquito virome includes plant viruses, such as Geminiviruses and Nanoviruses (Jones, 2003).

As described above, the SMS method significantly improves the detection of viruses in mosquitoes (Carissimo et al., 2016; Frey et al., 2016; Shi et al., 2018), allowing the identification of previously unknown entities, and the characterization of the virome of individual mosquitoes. Recent work supports the conclusion that mosquito virome is frequently dominated by specific ISVs. For instance, ISVs of the Flaviridae family account for 88.5% of the virome of *Culex* spp. mosquitoes from Mozambique (Cholleti et al., 2016). Similarly, ISVs are 88% of the virome of *Culex tritaeniorhynchus* from China (Shi et al., 2015). The virome of *Culex* spp. mosquitoes collected in different sites in Kenya and China was shown to differ both in terms of number and in relative abundance of arboviruses vs. ISVs (Atoni et al., 2018). Three known ISVs dominated the virome of wild-caught *Ae. aegypti* mosquitoes from Thailand and Australia: the phlebovirus PCLV (family *Bunyaviridae*), which represents >75% of the viral community in both sites; the unclassified Humaita-Tubiacanga virus (HTV), and the flavivirus Cell fusing agent virus (CFAV), which was previously found to be common in wild *Ae. aegypti* samples (Cook et al., 2006; Hall et al., 2017; Zakrzewski et al., 2018). The similarity of the virome in mosquitoes from Thailand and Australia contrasted with substantial differences in the composition and abundance of their bacterial community and mycobiota (Zakrzewski et al., 2018).

Similar to findings on the bacterial component of the mosquito microbiota, viral diversity is likely shaped by host- and environmental-related factor, including sex, diet, environmental temperature and ecological features of the resting sites (Atoni et al., 2018). For instance, arboviruses replicate at higher temperatures (i.e., 36.5–42°C in mammals and birds) than ISVs (i.e., around 28°C in tropical regions), supporting the idea that temperature is an important factor modulating viral prevalence and maintenance in mosquito field populations (Marklewitz et al., 2015).

The landscape of ISVs in the field as well as their prevalence in both laboratory and natural mosquito populations are still poorly described and require further investigation because ISVs may influence mosquito immunity, with effects on viral replication and gut microbiota diversity (Yamao et al., 2009; Blitvich and Firth, 2015; Bolling et al., 2015a, b; Hall et al., 2017; Souza-Neto et al., 2019).

## Microbiota as a Target for Novel Vector Control Strategies

**FIGURE 3:**
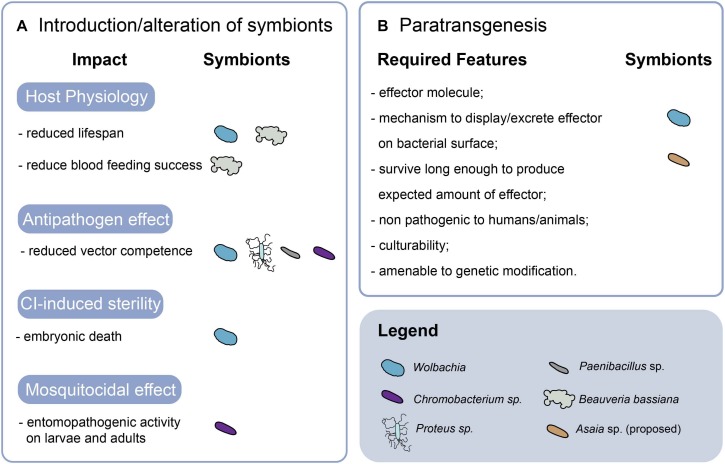
Microbiota-based approaches in vector control. The features and effects of strategies based on the introduction or alteration of symbionts **(A)**, and paratransgenesis **(B)** are summarized. CI, cytoplasmic incompatibility.

The increasingly emerging interactions among mosquito host, viral infection and microbiota are stimulating the development of strategies to exploit *Aedes* spp. microbiota for vector control. The application of microbiota in vector control include strategies that aim at altering or using microbiota *taxa* that were shown to have physiological impacts on the host or displaying mosquitocidal and antipathogen effects. Alternative strategies, collectively regarded as paratransgenesis, aim at interfering with pathogens via the genetic modification of endosymbionts to express antipathogen effector molecules in the mosquito host (Wang and Jacobs-Lorena, 2017). Figure 3 provides a summary of the currently available strategies.

### Introduction of Symbionts to Manipulate Host Physiology

Several strains of *Wolbachia pipientis* (Alphaproteobacteria, Rickettsiales) are able to manipulate host reproduction through Cytoplasmic Incompatibility (CI) (Werren et al., 2008). Establishment of a stable mosquito infection with a *Wolbachia* strain inducing CI can be exploited for mosquito control to induce sterility as a consequence of male release in the field (Incompatible Insect Technique, IIT) (Alphey et al., 2010). This strategy has been implemented also in *Ae. albopictus*, which naturally hosts two *Wolbachia* strains (*wAlbA* and *wAlbB*), generating a triple-infected strain with wPip from *Culex pipiens molestus* (Zhang et al., 2015). Field releases of *wPip Wolbachia*-infected *Ae. albopictus* males in Kentucky (United States) were shown to be effective in achieving a significant reduction in the number of females in the treated area (Mains et al., 2016) and large-scale population suppression trials are on the way in Guanzhou (China) (Mishra et al., 2018). The possibility to stably introduce *Wolbachia* in non-natural host species via transinfection opened new possibilities when *Wolbachia* infection was found to increase protection against arbovirues (Xi et al., 2005; Hedges et al., 2008; Teixeira et al., 2008). Specifically, when transfected in *Ae. aegypti*, the *Wolbachia w*MelPop-CLA strain reduces mosquito lifespan and its vector competence for DENV-2 and CHIKV (McMeniman et al., 2009; Moreira et al., 2009; Walker et al., 2011). After initial cage experiments, which showed that both *w*MelPop- and *w*Mel-infected *Ae. aegypti* could invade wild populations and reach high frequencies (McMeniman et al., 2009; Walker et al., 2011), field releases in Australia showed that *Wolbachia* could be established in mosquito populations (Hoffmann et al., 2011) and continue to reduce vector competence following establishment in the field (Frentiu et al., 2014). Since then, several small- and large-scale releases of transinfected *Ae. aegypti* are ongoing in several locations worldwide, including Indonesia, Vietnam, Australia, and Brazil (see World Mosquito Program, 2017; Dorigatti et al., 2018).

The application of *Wolbachia* for the control of *Ae. aegypti* mosquitoes in population replacement strategies stimulated investigation to assess the occurrence of *Wolbachia* in wild-caught mosquitoes, with contrasting results. *Wolbachia* was detected in wild *Ae. aegypti* larvae and adults from Florida and in adults from Thailand and the Philippines (Coon et al., 2016b; Thongsripong et al., 2017; Carvajal et al., 2018). In contrast, there was no evidence of *Wolbachia* in over 2,500 mosquitoes from the whole species range (Gloria-Soria et al., 2018). The presence of a natural *Wolbachia* infection in *Ae. aegypti* would be of great significance because a natural endosymbiont may circumvent the fitness loads related to the artificial mosquito-*Drosophila* system currently in use (Moreira et al., 2009; Schmidt et al., 2017; Gloria-Soria et al., 2018).

A pathogen enhancement effect with respect to DENV-2 was seen in *Ae. aegypti* re-infected with *Serratia odorifera*, opposite to the phenotype observed for *w*MelPop- and *w*Mel-infected *Ae. aegypti* (Apte-Deshpande et al., 2012, 2014). These findings emphasize the complex interplay among the host, the microbiota and the pathogens. These multifaced effects should also be considered in an ecological framework. For instance, it has been proposed that DENV inhibition of *w*MelPop- and *w*Mel-infected *Ae. aegypti* mosquitoes may be temperature-sensitive (Ye et al., 2016).

Other bacterial endosymbionts, such as *Spiroplasma* and *Arsenophonus* are capable of manipulating host reproduction (Briones et al., 2008; Duron et al., 2008; Terenius et al., 2008; Segata et al., 2016), suggesting that further exploration of microbiota in *Aedes* spp. mosquitoes may reveal additional candidates to be explored as tools for mosquito population control.

### Exploitation of Endosymbionts With Antipathogen Effects

A number of microbiota members were shown to have antipathogen activities (Blumberg et al., 2016). For example, some entomopathogenic fungi shorten mosquito life span or reduce blood feeding success (Kean et al., 2015). Analogously of *Wolbachia*, *Beauveria bassiana* influences mosquito vector competence by activating the Toll/Jak-Stat immune pathways in *Ae. aegypti* thus indirectly decreasing DENV-2 infection (Dong et al., 2012) and reducing vectorial capacity for ZIKV in *Ae. albopictus* (Deng et al., 2019). The presence of the ascomycota *Metarhizium anisopliae* was shown to correlate with reduced DENV-2 loads in *Ae. aegypti* females (Carballar-Lejarazú et al., 2008; Paula et al., 2011; Garza-Hernandez et al., 2013).

Insect-specific viruses appear to suppress arboviruses in mosquitoes not only through replicative interference due to their genetic similarity with arboviruses, but also by superinfection exclusion, a process whereby primary viral infections can block a secondary infection of a similar virus (Newman et al., 2011; Crockett et al., 2012; Kenney et al., 2014; Bolling et al., 2015b; Kuwata et al., 2015; Hall-Mendelin et al., 2016; Saldaña et al., 2017; Öhlund et al., 2019). So far, most studies involved *in vitro* systems and focused on IS-flaviviruses, with the exception of the IS-alphavirus Eilat (EILV) that could alter Sindbis virus titers *in vivo* (Nasar et al., 2015; Öhlund et al., 2019).

Isolation of cultivable bacteria from the midgut of field-collected *Ae. aegypti* mosquitoes from Panama and subsequent reintroduction of single-isolate bacterial species such as *Proteus* sp. and *Paenibacillus* sp. resulted in a significant decrease in DENV-2 infection (Ramirez et al., 2012). This effect was related to the transcriptional changes induced in a number of antimicrobial peptide genes in the midgut, including cecropin, gambicin, and attacin. In the same study, the authors identified a *Chromobacterium* sp. isolate that was later shown to be able to recolonize the gut of both *An. gambiae* and *Ae. aegypti* and block *Plasmodium* and DENV-2 infection, respectively (Ramirez et al., 2014). The Gram-negative *Chromobacterium* inhibits growth of other bacteria in the midgut, displays entomopathogenic activity on larvae and adults, and was suggested to exert *in vitro* and *in vivo* anti-pathogen activity through stable secondary metabolites. While romidepsin appeared to be the most likely *Chromobacterium*-produced metabolite responsible for antiplasmodial activity (Saraiva et al., 2018b), the anti-DENV activity is mediated by an aminopeptidase interfering with DENV-2 attachment by promoting the degradation of the *Flavivirus* E protein (Saraiva et al., 2018a). These effects on mosquitoes, together with its culturability, make *Chromobacterium* an ideal candidate to be integrated in strategies for controlling both mosquito populations and pathogen transmission.

One key aspect of these approaches is the feasibility of their use in field applications. Indeed, the capacity to spread efficiently in a wild population is essential. To do so, further research focused on the identification of selective pressures that could confer modified microbes an advantage over their wildtype counterparts is important and requires a better understanding of the physiological and genetic mechanisms favoring the presence of specific microbes among the community.

### Paratransgenesis Approaches

Paratransgenesis requires the identification of symbionts that can be isolated from host tissues and used for *in vitro* genetic transformation. Moreover, symbionts should show specific tissue tropism as the cycle of several pathogens initiates in the gut and ends with salivary gland, and vertical transmission to the progeny, thus allowing self-sustenance of the modified symbionts in the field (Mancini et al., 2018). Moreover, such symbionts have to be well established in the mosquito host in order to survive long enough to produce the effectors in the necessary amounts and display or excrete the effector molecule on their surface (Wilke and Marrelli, 2015).

*Asaia* emerged as a promising candidate for the paratransgenic-based control of malaria, as it was shown to be important for larval development in *Anopheles* spp., can be genetically manipulated, can be easily acquired by mosquitoes and it is vertically transmitted (see Saldaña et al., 2017 for a review). This bacterium has been detected in both laboratory and field mosquitoes, in both *Ae. albopictus* and *Ae. aegypti* (Chouaia et al., 2010; Minard et al., 2013a). *Asaia* was already modified to secrete anti-malaria molecules (Bongio and Lampe, 2015) and the results of semi-field trials suggested it can rapidly spread in wild populations of *An. stephensi* and *An. gambiae* (Mancini et al., 2016). *Wolbachia* and *Asaia* appear to negatively compete, with *Asaia* occurrence in *Wolbachia*-infected mosquito species being low (Rosso et al., 2018). Thus, a potential use of *Asaia* in paratransgenic approaches to control *Aedes* spp. mosquitoes will require a better understanding of the dynamic interactions between these two endosymbionts in the field.

## Conclusion

The possibility to rear mosquitoes in which a particular bacterial species is dominant among the gut microbiota supports the development of strategies based on symbionts that induce antiviral responses or antiviral molecules in *Aedes* spp. (Baldacchino et al., 2015; Ritchie et al., 2018). Achieving a deeper understanding of the molecular mechanisms underlying the interaction between microbiota and pathogens may also lead to the selection of mosquito strains resistant to infection. On this basis, it is important to further expand our understanding of the physiological and metabolic interactions between *Aedes* spp. mosquitoes and their microbiota, in particular providing consistent answers to key questions, such as: (i) what is the composition of *Aedes* spp. microbiota in the field? (ii) what is the level of its variability and which are the parameters affecting such differences? (iii) how are community members of *Aedes* spp. microbiota transmitted cross-generationally? (iv) how do endosymbionts released into the environment compete with the natural microbiota members of mosquitoes?

Besides having practical applications, these questions will also shed new light on the establishment and maintenance of symbiotic interactions. Interestingly, apart from *Wolbachia*, the bacterial species that have been identified so far to contribute to vector competence (i.e., *Serratia* and *Chromobacterium*) are sporadically detected in field mosquitoes supporting the hypothesis that while the core microbiota may contribute to mosquito physiology, rare and differentially distributed bacterial species should be more carefully studied in relation to vector competence.

## Author Contributions

MB and FS conceived, designed, and wrote the manuscript. FS prepared the figures. MC critically reviewed the manuscript and contributed to figure improvement. All authors approved the final version of the manuscript.

## Conflict of Interest Statement

The authors declare that the research was conducted in the absence of any commercial or financial relationships that could be construed as a potential conflict of interest.
